# Retraction: The interactive effect of pH variation and cadmium stress on wheat (*Triticum aestivum* L.) growth, physiological and biochemical parameters

**DOI:** 10.1371/journal.pone.0284069

**Published:** 2023-04-19

**Authors:** The PLOS ONE Editors

The *PLOS ONE* Editors retract this article [1] because it was identified as one of a series of submissions for which we have concerns about authorship, competing interests, and peer review. We regret that the issues were not addressed prior to the article’s publication.

SUR, QX, US, MAB, and AR did not agree with the retraction. LR, GY, ANS, MSJ, AD, and ZD either did not respond directly or could not be reached.
